# Single-Cell Analysis of the Antimicrobial and Bactericidal Activities of the Antimicrobial Peptide Magainin 2

**DOI:** 10.1128/spectrum.00114-22

**Published:** 2022-07-13

**Authors:** Farzana Hossain, Md Masum Billah, Masahito Yamazaki

**Affiliations:** a Nanomaterials Research Division, Research Institute of Electronics, Shizuoka University, Shizuoka, Japan; b Integrated Bioscience Section, Graduate School of Science and Technology, Shizuoka University, Shizuoka, Japan; c Department of Physics, Faculty of Science, Shizuoka University, Shizuoka, Japan; University of Manitoba

**Keywords:** antimicrobial peptides, *Escherichia coli*, bactericidal activity, cell death, cell permeabilization, cell proliferation, confocal microscope, interaction time, microcolony, plasma membrane, single-cell analysis

## Abstract

Antimicrobial peptides (AMPs) inhibit the proliferation of or kill bacterial cells. To measure these activities, several methods have been used, which provide only the average value of many cells. Here, we report the development of a method to examine the antimicrobial and bactericidal activities of AMPs at the single-cell level (i.e., single-cell analysis) and apply this strategy to examine the interaction of an AMP, magainin 2 (Mag), with Escherichia coli cells. Using this method, we monitored the proliferation of single cells on agar in a microchamber and measured the distribution of the number of cells in each microcolony using optical microscopy. For method A, we incubated cells in the presence of various concentrations of AMPs for 3 h. The fraction of microcolonies containing only a single cell, *P*_single_, increased with the Mag concentration and reached 1 at a specific concentration, which corresponded to the MIC. For method B, after the interaction of a cell suspension with an AMP for a specific time, an aliquot was diluted to stop the interaction, and the proliferation of single cells then was monitored after a 3-h incubation; this method permits the definition of *P*_single_(*t*), the fraction of dead cells after the interaction. For the interaction of Mag with E. coli cells, *P*_single_(*t*) increased with the interaction time, reaching ~1 at 10 and 20 min for 25 and 13 μM Mag, respectively. Thus, these results indicate that a short interaction time between Mag and E. coli cells is sufficient to induce bacterial cell death.

**IMPORTANCE** To elucidate the activity of antimicrobial peptides (AMPs) against bacterial cells, it is important to estimate the interaction time that is sufficient to induce cell death. We have developed a method to examine the antimicrobial and bactericidal activities of AMPs at the single-cell level (i.e., single-cell analysis). Using this method, we monitored the proliferation of single cells on agar in a microchamber and measured the distribution of the number of cells in each microcolony using optical microscopy. We found that during the interaction of magainin 2 (Mag) with E. coli cells, the fraction of dead cells, *P*_single_(*t*), increased with the interaction time, rapidly reaching 1 (e.g., 10 min for 25 μM Mag). This result indicates that Mag induces cell death after a short time of interaction.

## INTRODUCTION

Patients suffering from certain diseases must take antibiotics to inhibit the proliferation of or kill bacterial cells. Notably, seriously ill people may require many antibiotics to recover. For such patients, infection with multidrug-resistant (MDR) bacteria, which cannot be killed even using multiple antibiotics, may be fatal (1, 2). It is therefore important to develop new types of antibiotics to prevent infection by MDR bacteria. Some antimicrobial peptides (AMPs) are candidates for these new types of antibiotics (3–5), given that AMPs are effective, broad-spectrum antibiotics and potentially novel therapeutic agents. AMPs produced by various organisms (e.g., mammals [including humans], amphibians, insects, plants, and prokaryotes) suppress the proliferation of or kill Gram-negative and -positive bacteria and fungi (3, 6–10). Most AMPs are small peptides with masses below 50 kDa, though some are larger (11). AMPs adopt various structures, such as α-helices (12–14), β-strands (15), or extended or random structures (16, 17). To date, more than 3,000 AMPs have been found and isolated (18). AMPs act in various manners, including damaging the plasma membranes of bacterial cells (19, 20) and entering the cytoplasm (21, 22) to bind to DNA and/or important proteins (23).

MIC and minimum bactericidal concentration (MBC) values are used to express the antimicrobial activity and bactericidal activity (respectively) of antimicrobial compounds such as antibiotics, AMPs, and antibacterial drugs (24, 25). The MIC of an antimicrobial compound typically is defined as the minimum concentration of the compound that inhibits the proliferation of bacteria growing in suspension. The MBC of an antimicrobial compound typically is defined as the minimum concentration required to kill more than 99.9% of the initial viable bacterial cells. Both the MIC and MBC are determined only by the concentration of the antimicrobial compound and do not provide information on the interaction time required for the AMP to induce bacterial cell death. In contrast, in the time-kill assay, after the interaction of an antimicrobial compound with bacterial cells in suspension for a specific time, the density of live cells (CFU/mL) is measured by visually counting the number of colonies after incubation for 24 h (or longer) on an agar plate (24, 25). Time courses of the fraction of viable cells are obtained for various concentrations of the antibiotic. Hence, the time-kill assay provides information on the effect of time and the concentration of the antimicrobial compound on its antimicrobial activity, with the bactericidal activity being defined by a 99.9% decrease in viable cells (CFU/mL). All these methods use a suspension of bacterial cells; thus, only the average value of the parameter across of many cells (i.e., the ensemble average of values) is obtained. Moreover, these methods are time-consuming, given that measurements typically require more than 24 h.

In the present report, we describe the development of a method to determine the antimicrobial and bactericidal activities of AMPs at the single-cell level (i.e., a single-cell analysis of these activities). This method monitors the proliferation of single cells on the agar in a microchamber, permitting measurement of the number of cells per microcolony using optical microscopy. Using this method, we examined the interaction time required to induce bacterial death in Escherichia coli cells in the presence of an AMP, magainin 2 (Mag). Here, the interaction time, which is the same as the experimental time of incubation of cells with an AMP solution, is regarded as the time required for the most important steps in a given AMP’s bactericidal activity (e.g., rapid cytoplasmic leakage). Mag was selected as the AMP for validating this method because Mag is known to induce pore formation in the membranes of lipid vesicles, inducing leakage of internal contents from the vesicles, and the mechanism of pore formation by Mag is well understood (26–33). To prevent the interaction of AMPs and antibiotics with the unknown substances existing in most media, we used a synthetic medium (EZ medium) in this method (20). As a control experiment, we examined the activities of an antibiotic, tetracycline, using this analysis.

## RESULTS AND DISCUSSION

### MIC against E. coli.

First, we investigated the antimicrobial activity of Mag and tetracycline using a standard method (24, 25) and determined that the MICs of Mag and tetracycline against E. coli K-12 (NBRC 3301) in EZ medium (final cell density, ~1 × 10^5^ CFU/mL) were 25 and 16 μM, respectively.

### Growth curve of cells into a microcolony from a single cell.

**FIG 1:**
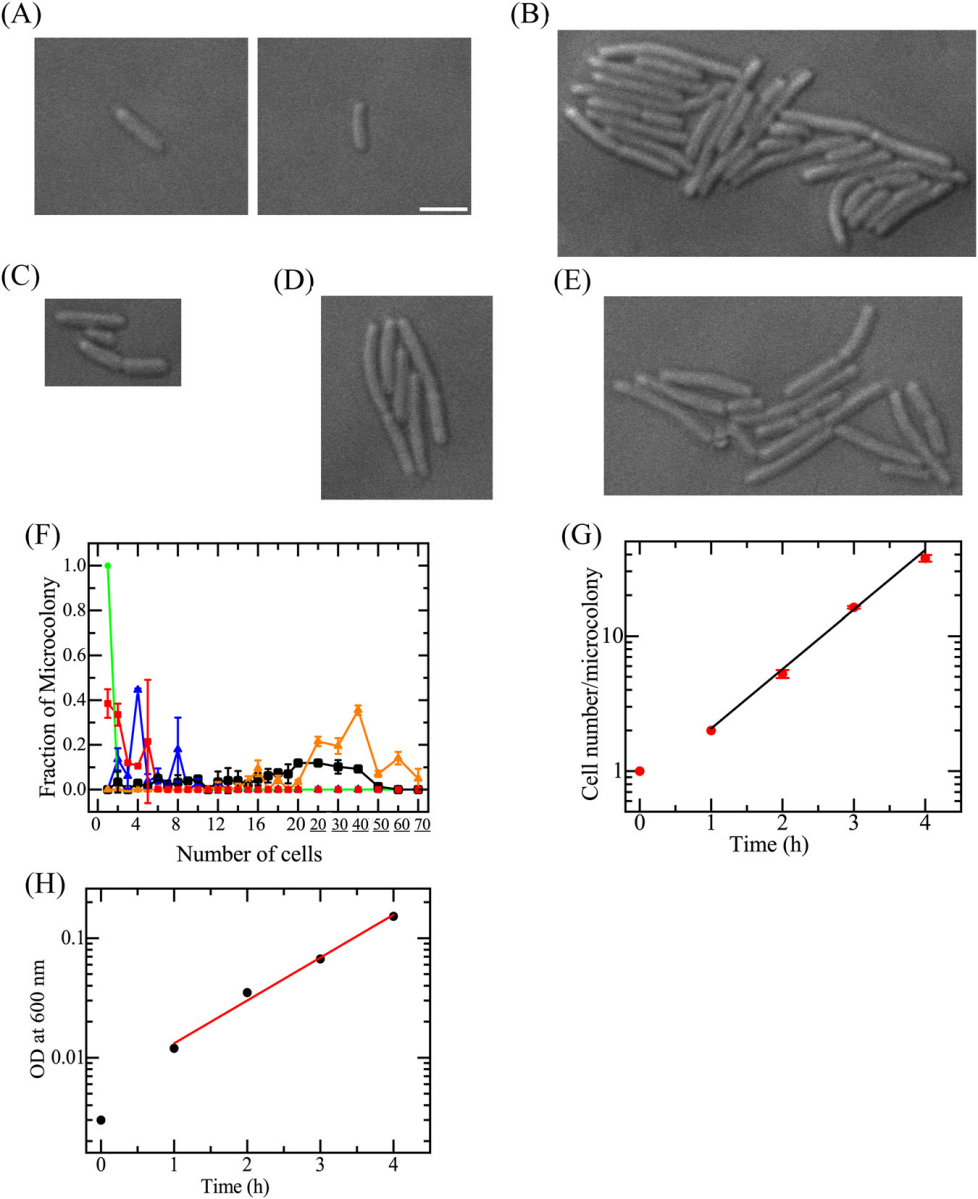
Proliferation of single E. coli cells on an agar medium in a chamber. (A to E) DIC images of microcolonies starting from single cells after incubation at 37°C for 0 h (A), 4 h (B), 1 h (C), 2 h (D), and 3 h (E). Bar, 2 μm. (F) Distribution of the number of cells per microcolony in a chamber after incubation for 0 h (green ●), 1 h (red ■), 2 h (blue ▴), 3 h (black ■), and 4 h (orange ▴). On the *x* axis, 20, 30, 40, 50, 60, and 70 indicate 21 to 29 cells, 30 to 39 cells, 40 to 49 cells, 50 to 59 cells, 60 to 69 cells, and 70 to 79 cells, respectively. The error bars denote the SD values (*N *= 2). (G) Time course of the average number of cells per microcolony, *N*(*t*). (H) Time course of the turbidity of an E. coli cell suspension. The absorbance at 600 nm (OD_600_) was measured.

We examined our system of bacterial cell culture in a handmade microchamber (“chamber”). An aliquot (10 μL) of E. coli cell suspension in EZ medium was spread onto the agar in the chamber. The chamber was then covered with a coverslip, and the cells were observed using differential interference contrast (DIC) microscopy. Figure 1A shows two E. coli cells on agar medium in the chamber before incubation (*t *= 0), indicating that each cell was well separated on the agar plate. Then, E. coli cells in the chamber were incubated at 37°C for 4 h. Figure 1B shows a typical DIC image of E. coli cells after 4 h of incubation. In this image, 36 cells formed a microcolony, and many such microcolonies were observed. Here, a microcolony was defined as an aggregate of several cells originating from a single mother cell. The mean ± standard deviation (SD) of the number of cells in a microcolony was 36 ± 18 (*n *= 173, where *n* is the number of microcolonies observed).

We monitored the proliferation of cells in each microcolony by observing the E. coli cells in the chamber after incubation at 37°C for various times (Fig. 1C to E). Figure 1F shows the distribution of the number of cells per microcolony. For each incubation time, there was a wide distribution in the number of cells per microcolony, and the number of cells at the peak of the distribution increased with time. Thus, all cells in all microcolonies continued to proliferate, and the mean number of cells in a microcolony increased with time. We performed two independent experiments (*N *= 2) and determined *N*(*t*), the mean (and SD) of the number of cells per microcolony after an incubation time of *t* (Fig. 1G). The time course of *N*(*t*), i.e., the growth curve of single-cell microcolonies, was determined as

(1) $$N\left(t\right)=N_{0}\times 2^{t/\tau }=2^{t/\tau }$$

where *τ* is the generation time, and *N*_0_ is the number of cells per microcolony at *t *= 0; for this experiment, *N*_0_ = 1. As shown in Fig. 1G, the growth curve from 1 to 4 h was well fitted by a straight line, indicating that the cells were in log phase. Based on the slope (*A*) of this line, we calculated that the generation time *τ* was 41 ± 2 min, given that *τ* = log_10_ 2/*A*. We separately calculated the generation time using the standard method (i.e., analysis of the time course of absorbance of a suspension of cells). Figure 1H shows the growth curve of E. coli cells in suspension obtained by absorbance measurements. The absorbance at 600 nm, OD_600_, is due to the light scattering from the cell suspension, and thus, the OD_600_ value is proportional to the cell density in a suspension. This method provided a *τ* value of 50 min (mean ± SD, 53 ± 4 min; *N *= 2). Therefore, the doubling time of E. coli cells obtained with the standard method using the turbidity of the cell suspension was similar to that obtained using analysis of the growth curve of cells in microcolonies in the chamber.

Here, we consider the distribution of the number of cells per microcolony after the incubation of single cells for a specific time. Figure 1F shows a wide distribution of cell numbers for each incubation time, but this distribution shifts with higher cell numbers. To consider this distribution, we calculated a theoretical distribution of the number of cells per microcolony based on a simple model (for details, see section S1 and Fig. S1 in the supplemental material). Based on this model, the distributions of the number of cells per microcolony at all incubation times were sharp; after 2- and 4-h incubation periods, all microcolonies had the same number of cells (i.e., 8 and 64 cells for 2- and 4-h incubations, respectively), and after 1- and 3-h incubations, there were only 2 patterns for the number of cells per microcolony (i.e., 2 and 4 cells after a 1-h incubation, 16 and 32 cells after a 3-h incubation). Thus, the experimental distribution of the number of cells per microcolony (Fig. 1F) had a much wider distribution of the number of cells for each incubation time than the theoretical distribution (Fig. S2). This comparison indicates that the generation time of E. coli cells fluctuated, even though all possessed the same genomic sequence. The difference in generation time among single E. coli cells presumably was attributable to the stochasticity of the metabolic reactions and consequent changes in cell cycle and the increase in cell length at the single-cell level (34, 35).

### Single-cell analysis of the antimicrobial activity of magainin 2.

Next, we investigated the effects of Mag on the proliferation of single E. coli cells in the chamber. Two single-cell analysis methods were used to detect the antimicrobial and bactericidal activities of AMPs. Here, we used method A to determine the antimicrobial activity of AMPs. Immediately after mixing an E. coli suspension into EZ medium with various concentrations of AMP (here, Mag) solution in EZ medium (final cell density, 1.6 × 10^4^ CFU/mL), 10 μL of this suspension was transferred onto agar medium in a chamber and incubated at 37°C for 3 h; we then measured the number of cells per microcolony. At 0 h, all cells were separately located on the agar medium (i.e., each microcolony comprised a single cell), similar to the cells without interaction with AMPs on the agar medium described above (e.g., Fig. 1A).

**FIG 2:**
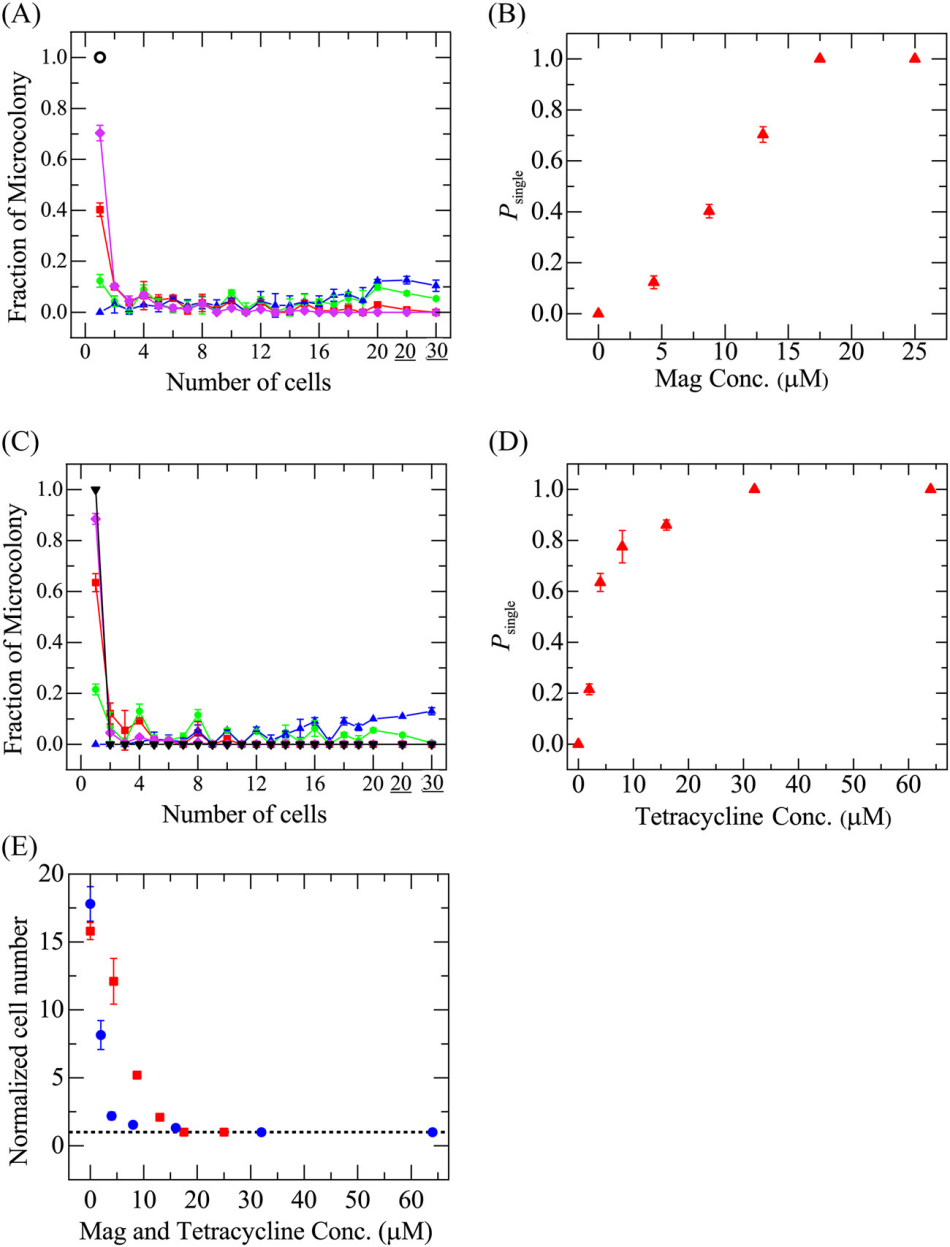
Effect of Mag and tetracycline on the proliferation of single E. coli cells in the single-cell analysis (method A). E. coli cells in the chamber were incubated in the presence of various Mag and tetracycline concentrations for 3 h. (A) Distribution of the number of cells per microcolony after incubation with Mag concentrations of 0 μM (blue ▴), 4.4 μM (green ●), 8.8 μM (red ■), 13 μM (purple ♦), and 25 μM (black ○). (B) Mag concentration dependence of *P*_single_. For panels A and B, the mean values and SDs of the fraction of microcolonies and *P*_single_ (*N *= 3) are shown. (C) Distribution of the number of cells per microcolony after 3 h of incubation with tetracycline concentrations of 0 μM (blue ▴), 2 μM (green ●), 4 μM (red ■), 16 μM (purple ♦), and 32 μM (▾). For panels A and C, 20 and 30 mean 21 to 29 cells and 30 to 39 cells, respectively. (D) Tetracycline concentration dependence of *P*_single_. For panels C and D, the mean values and SDs of *P*_single_ (*N *= 2) are shown. (E) Mag (red ■) and tetracycline (blue ●) concentration dependence of the normalized total number of cells per chamber. The mean values and SDs are shown. The dashed line denotes the line of the normalized cell number of 1. Conc, concentration.

Figure 2A shows the distribution of the number of cells per microcolony after a 3-h incubation. In the presence of 25 μM Mag, only single cells were observed in all 158 microcolonies, whereas in the absence of Mag, the mean number of cells per microcolony was 16. This result indicated that no cell proliferation occurred in the presence of 25 μM Mag. On the other hand, Mag concentrations at and below 13 μM resulted in various numbers of cells per microcolony (ranging from 1 to 39). Figure 2B shows that the fraction of microcolonies containing only a single cell, *P*_single_, after a 3-h incubation increased with an increasing Mag concentration and reached 1 at and above 18 μM. This result shows that no single cells proliferated at or above 18 μM Mag, indicating that the number of total cells in the chamber did not increase. Therefore, the minimum concentration needed to reach a *P*_single_ value of 1 (18 μM) corresponded to the MIC determined using single-cell analysis method A and was similar to that determined using the standard MIC measurement method (25 μM).

Here, we consider the physiological meaning of *P*_single_ obtained using method A for the single-cell analysis. Since a cell divides into two cells after a generation time *τ*, the number of cells per microcolony after a time of *nτ* is 2*^n^* if all cells are alive. When we mix an AMP solution with a cell suspension, the cells are at various stages of growth; some cells have just been produced by cell division, whereas some cells are about to divide (see section S1). Thus, the cells do not divide synchronously. If the number of cells per microcolony after a 3-h incubation with AMPs is 1, the single cells cannot divide into two cells during the first-generation interval in any of the microcolonies, even if the cells are septating before mixing with the AMP solution, indicating that cell proliferation has been inhibited starting from the initiation of interaction with the AMP. Therefore, *P*_single_ is the fraction of cells (among all initial cells) that stop proliferating within the first-generation interval; if *P*_single_ = 1, then the proliferation of all cells has been inhibited. The results in Fig. 2B indicate that at and above concentrations of 18 μM Mag, all cells stopped proliferating within the first-generation interval (i.e., 41 min) after the initiation of interaction with Mag.

As a control experiment, we investigated the antimicrobial activity of a bacteriostatic antibiotic, tetracycline, using method A. Tetracycline impedes proliferation by binding to the 30S ribosomal subunit to inhibit protein synthesis (36, 37). Figure 2C shows the tetracycline concentration dependence of the distribution of the number of cells per microcolony after a 3-h incubation. *P*_single_ increased with an increasing tetracycline concentration, reaching 1 at 32 μM (Fig. 2D). Thus, this concentration (32 μM) corresponded to the MIC as determined using single-cell analysis; this value was 2-fold that determined using the standard measurement method (16 μM). At 16 μM tetracycline, *P*_single_ was 0.86, and the number of cells per microcolony ranged from 1 to 8, indicating that all cells ceased proliferation within four generations after the start of interaction with tetracycline.

Using the data obtained with single-cell analysis (method A), we determined the total number of cells in a chamber after a 3-h incubation with various concentrations of Mag; the total number was calculated by summing the product of the number of cells per microcolony and the number of microcolonies with a given number of cells. The numbers of cells in each chamber at the initial time were not identical; therefore, we normalized the total cell number in a given chamber by dividing the total number of cells in that chamber after a 3-h incubation by the number of cells in that chamber at *t *= 0, which equals the number of microcolonies there. In effect, the normalized cell number is the amplification factor from the initial number of cells due to cell proliferation. Figure 2E shows that at and above 18 μM Mag, the normalized cell number was 1, indicating no proliferation, but at and below 13 μM Mag, the normalized cell number increased as the Mag concentration decreased, indicating that the rate of cell proliferation decreased with an increasing Mag concentration, reaching zero above the threshold concentration. Figure 2E is similar to a figure, which was employed to determine the MIC value using the standard MIC measurement method (i.e., absorbance vs AMP concentration). Thus, the result shown in Fig. 2E further supports the proposal that 18 μM Mag corresponds to the MIC under these conditions. The tetracycline results showed that at and above 32 μM tetracycline, the normalized cell number was 1, indicating no proliferation, but at and below 16 μM tetracycline, the normalized cell number increased with decreases in tetracycline concentration (Fig. 2E). This result supports our inference that 32 μM tetracycline corresponds to the MIC under these conditions.

The MIC typically is defined as the minimum concentration that prevents significant growth of cells after ~24 h of interaction, i.e., no significant increase in turbidity of the cell suspension as detected by the unaided eye (38). Using single-cell analysis (method A), we can define the “single-cell MIC” as the minimum concentration of an AMP or antibiotic that is sufficient to inhibit completely the proliferation of cells, resulting in no increase in cell number for microcolonies growing on an agar plate. To satisfy this condition, the proliferation of cells must stop within the first generation of all cells after the start of interaction with the AMP or antibiotic. In this sense, the minimum concentration to reach a *P*_single_ of 1 determined using single-cell analysis equals the value of the single-cell MIC, which appears to correspond to the MIC determined using the standard method. The small difference between the MIC values obtained using single-cell analysis and those using the standard MIC measurement may be due to differences in the condition of cells (see section S2 for details).

### Effect of interaction time of Mag with E. coli cells on its bactericidal activity.

To elucidate the mechanism of Mag-induced bactericidal activity, we used single-cell analysis (method B) to investigate the effect on cell death of the interaction time of Mag with E. coli cells. Using method B, after the interaction of an AMP (here, Mag) with cells in EZ medium for a specific interval, the cell suspension was diluted with fresh EZ medium, ensuring that the final AMP concentration was low enough that AMP no longer had an antimicrobial effect. Following dilution, an aliquot (10 μL) of the diluted suspension was transferred onto agar medium in a chamber and incubated at 37°C for 3 h, and the number of cells per microcolony then was measured.

**FIG 3:**
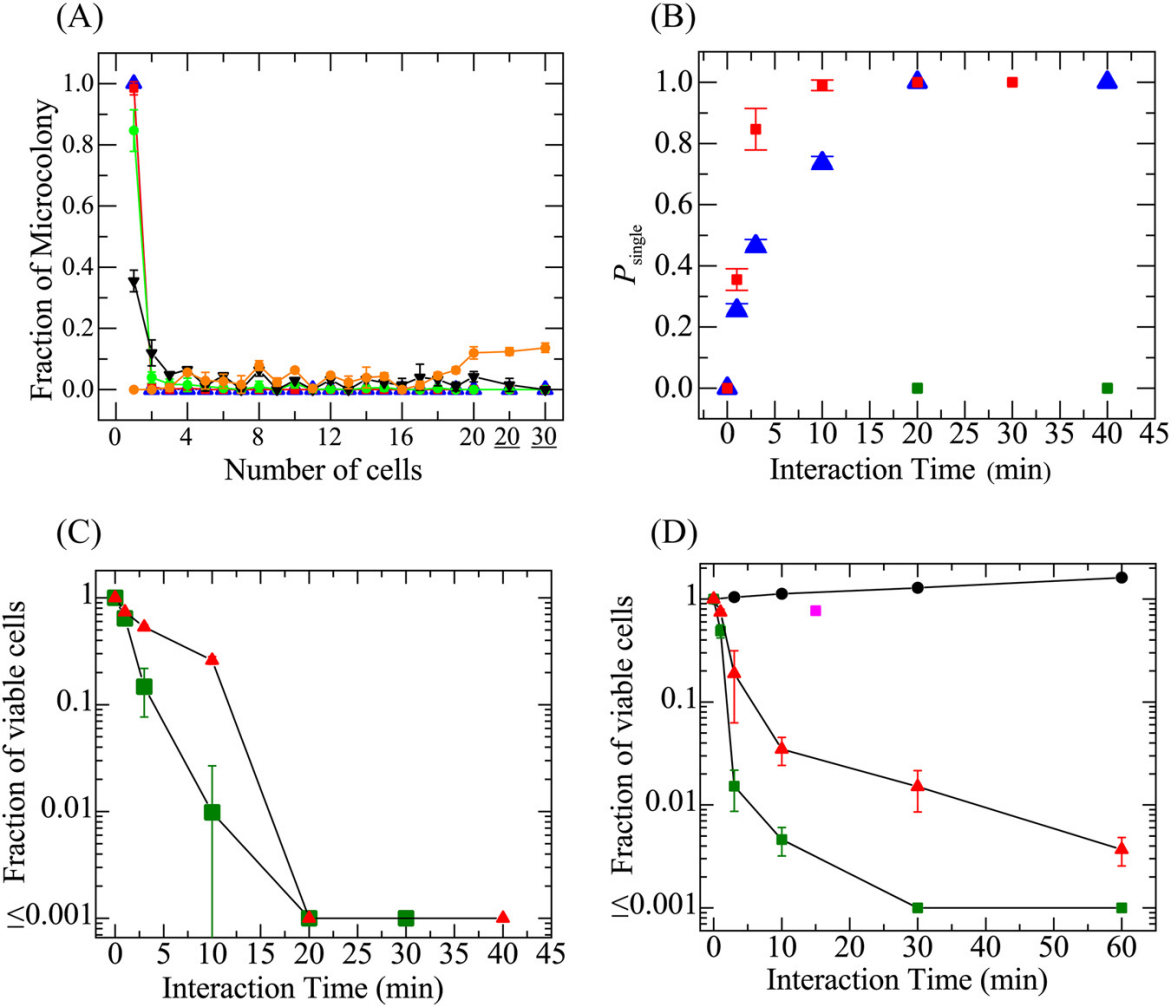
Effect of interaction time of Mag with E. coli cells on its bactericidal activity. A cell suspension in EZ medium was mixed with Mag solution or tetracycline solution in EZ medium and incubated for a specific time, then the suspension was diluted sufficiently with fresh EZ medium. After 3 h of incubation in the chamber, we counted the number of cells per microcolony. (A) Distributions of the number of cells per microcolony after interaction with 25 μM Mag for 0 min (orange ●), 1 min (▾), 3 min (green ●), 10 min (red ■), and 30 min (blue ▴). On the *x* axis, 20 and 30 mean 21 to 29 cells and 30 to 39 cells, respectively. The mean values and SDs of the fraction of microcolonies (*N *= 3) are shown. (B) Dependence of *P*_single_ on the interaction time with 25 μM (red ■) and 13 μM (blue ▴) Mag. The mean values and SDs of *P*_single_ were obtained by conducting 3 independent experiments for each interaction time using 120 to 200 microcolonies. As a comparison, the results for tetracycline (i.e., the dependence of *P*_single_ on the interaction time with 16 μM tetracycline) are shown (green ■). For tetracycline, the mean values of *P*_single_ were obtained by conducting 2 independent experiments for each interaction time using 120 to 200 microcolonies. (C) Time course of the fraction of viable cells obtained using the single-cell analysis shown in panel A for interaction with 25 μM (green ■) and 13 μM (red ▴) Mag. (D) Time course of the fraction of viable E. coli cells obtained using the time-kill assay for interaction with 25 μM (green ■), 13 μM (red ▴), and 0 μM (black ●) Mag. The fraction of viable cells in the absence of membrane potential is also shown (magenta ■) for 100 μM CCCP and 0 μM Mag (this result is cited with permission from reference 22). The mean values and SDs of the fraction of viable cells (*N *= 3) are shown in panels C and D.

First, we examined the effect on E. coli cells of the interaction time with 25 μM Mag (i.e., the MIC determined using the standard measurement). Of 145 total microcolonies obtained following a 3-min interaction of 25 μM Mag with E. coli cells in a suspension (final cell density, 1.7 × 10^5^ CFU/mL), 112 microcolonies comprised single cells; the other colonies consisted of 2 to 18 cells (Fig. 3A). Thus, *P*_single_ was 0.77. The mean ± SD of *P*_single_ (*N* = 3) under the same conditions was 0.84 ± 0.06. Figure 3A shows the distribution of the number of cells per microcolony for various interaction times. For 10- and 30-min interaction times, the *P*_single_ values were 0.99 and 1.0, respectively. Here, *P*_single_ = 1.0 meant that all microcolonies comprised only single cells. Figure 3B (red ■) shows the dependence of *P*_single_ on the interaction time with 25 μM Mag. *P*_single_ increased with time, reaching ~1 at 10 min. Next, we examined the effect of 13 μM Mag. *P*_single_ increased with time, reaching 1 at 20 min (Fig. 3B, blue ▴).

To examine the effect of the incubation time on the *P*_single_ value, we incubated the cells in a chamber for 24 h and found that *P*_single_ = 1.0 for both 10- and 20-min interactions with 25 μM Mag (*N *= 2), results that were almost the same as those obtained after a 3-h incubation. After a 3-min interaction, some microcolonies grew significantly and overlapped each other, preventing the accurate determination of *P*_single_. These results indicate that there is no long postantibiotic effect (39, 40) for Mag against E. coli cells, an inference consistent with the results obtained using the standard time-kill assay with a 24-h incubation (see details below and in Fig. 3D).

After the interaction of Mag with E. coli cells for a specific time, we diluted the cell suspension with fresh medium, terminating the interaction of Mag with the cells. If the cells simply were to stop proliferating without dying during the interaction with Mag, they would resume proliferation after dilution (i.e., reversible inhibition of cell proliferation). In contrast, if the cells died during the interaction, they would not resume proliferation after dilution (i.e., irreversible inhibition of cell proliferation and thus, cell death). In the above single-cell experiments, the values of *P*_single_ indicate the fraction of cells whose proliferation is inhibited irreversibly, i.e., the fraction of dead cells. Therefore, Fig. 3B indicates that the minimum interaction times to kill all E. coli cells were 10 and 20 min for 25 and 13 μM Mag, respectively.

We compared the results obtained using single-cell analysis (method B) with those obtained using the standard time-kill assay (24, 25). For this purpose, we estimated the viable cell density (CFU/mL) after interaction with Mag for specific intervals of time. For method B, we define live cells as the cells in microcolonies containing 2 cells or more. As an example, we consider the viable cell density after the interaction of 25 μM Mag with E. coli cells for 3 min. Following the interaction, 10 μL of a 10-fold diluted cell suspension was incubated for 3 h using the single-cell analysis; 33 microcolonies comprised more than 2 cells among a total of 145 microcolonies, indicating that 33 cells were alive in this suspension. Thus, the viable cell density was determined to be 3.3 × 10^4^ CFU/mL. Since the initial number of viable cells before the interaction with Mag equals the number of microcolonies in the same chamber, the initial viable cell density was determined to be 1.45 × 10^5^ CFU/mL. The fraction of viable cells (*P*_live_) here is defined as the ratio of the viable cell density of a cell suspension incubated in the presence of Mag for a specific period, compared to that of the initial cell suspension. Therefore, the *P*_live_ value after a 3-min interaction with 25 μM Mag was 0.23. The *P*_live_ value also can be obtained as 1 − *P*_single_, given that *P*_single_ is the fraction of dead cells. Under the same conditions, we obtained a *P*_single_ value of 0.77; thus, *P*_live_ = 0.23, which agrees with the value obtained using the standard definition described above. Another example is the 30-min interaction with 25 μM Mag, where all microcolonies comprised only a single cell (i.e., *P*_single_ = 1), indicating that *P*_live_ = 0. Figure 3C shows the effect of the interaction time on the *P*_live_ value obtained using single-cell analysis, indicating that *P*_live_ decreased with the interaction time, reaching 0.01 and 0.3 after 10 min at Mag concentrations of 25 and 13 μM, respectively, and reaching 0 after 20 min for both Mag concentrations. Next, we estimated the experimental error of the fraction of viable cells (CFU/mL) determined using this single-cell analysis, which is due to the limited number of examined cells at the current stage of the analysis. When *P*_live_ = 0 (i.e., the number of microcolonies comprising 2 cells or more is 0), the error originates from the possible presence of a microcolony containing more than 2 cells (i.e., a live cell in the chamber). The presence of a viable cell on the agar plate means that the viable cell density is 1,000, and thus, its *P*_live_ value is 5.0 × 10^−3^ if there are 200 microcolonies, which corresponds to the error of *P*_live_.

In contrast, for the time-kill assay, we diluted a cell suspension more than 10-fold after interaction with Mag for a specific time, incubated the cells on agar in a petri dish for 24 h at 37°C, and then counted the number of colonies (i.e., the macrocolonies) to obtain the number of live cells. We performed a time-kill assay for the interaction of 25 and 13 μM Mag with E. coli cells (final cell density, 1.1 × 10^6^ CFU/mL). Figure 3D shows that the fraction of viable cells (*P*_live_) in the suspension decreased with increasing interaction time with Mag. For a 30-min interaction with 25 μM Mag, more than 99.9% of the cells were dead. For interaction with 13 μM Mag, *P*_live_ decreased gradually, and 99.6% of the cells were dead after 1 h. The *P*_live_ values for interactions less than 10 min (obtained using single-cell analysis method B) were slightly larger than those obtained using the time-kill assay, but for 30-min interactions, both methods provided a *P*_live_ value of less than 0.001 (i.e., more than 99.9% of the cells were dead). For the time-kill assay, the number of live cells after interaction with AMP for specific times is defined based on the number of visible macrocolonies, and thus, the small microcolonies (which cannot be observed with the unaided eye) are not counted as live cells, whereas microcolonies containing 2 cells or more can be counted as live cells for the single-cell analysis. This difference is the main reason why the *P*_live_ values obtained using single-cell analysis were larger than those obtained using the time-kill assay. These small microcolonies (containing 2 cells or more) in the time-kill assay are produced by the live cells after interaction with AMP, but after a 24-h incubation, these microcolonies do not become visible due to the decrease in the rate of proliferation and the death of cells. The time-kill assay does not detect such live cells due to its limited sensitivity. Therefore, in this sense, the single-cell analysis is a higher-sensitivity method for detecting live cells than the time-kill assay.

In conclusion, using the single-cell analysis (method B), we succeeded in defining the minimum interaction time for the bactericidal activity of Mag (i.e., the minimum time when *P*_single_ becomes 1), indicating that for Mag, a short interaction is sufficient to induce the death of bacterial cells.

Membrane potential (Δ*φ*) plays an important role in various physiological phenomena (20, 41, 42). In Fig. 3D, we also show the time-kill assay result for cells exposed to 100 μM carbonyl cyanide *m*-chlorophenylhydrazone (CCCP), a protonophore (22), in the absence of an AMP. After a 15-min interaction of 100 μM CCCP with E. coli cells, the fraction of viable cells decreased by only 23%. A high concentration of CCCP is known to decrease greatly the Δ*φ* of the bacterial plasma membrane (41). Therefore, this result indicates that a loss of Δ*φ* for 15 min does not induce significant cell death. The loss of Δ*φ* inhibits the proliferation of cells due to the suppression of cell division (41), but this effect is reversible, i.e., after CCCP is removed, Δ*φ* is restored, and cell proliferation reinitiates. The small fraction of cell death (23%) may reflect the decrease in Δ*φ* or the effect of other factors (e.g., growth phase and acidic pH) (43). This result and the data shown in Fig. 3B indicate that Δ*φ* has a small effect on cell viability compared to that of Mag.

As a control experiment, we investigated the bactericidal activity of tetracycline using method B. We examined the number of cells per microcolony after interaction with 16 μM tetracycline for various times (0 to 40 min). For all conditions, *P*_single_ = 0 (Fig. 3B), indicating that after dilution, the cells restarted proliferation, irrespective of the incubation time. Together, the results shown in Fig. 3B and 2D demonstrate that tetracycline is a bacteriostatic antibiotic.

Wong et al. have reported a method for observing bacterial cells using a microfluidic chamber (44, 45). Their setup may allow more readily for the dilution of AMP solution and longer incubation, which would be useful for the single-cell analysis proposed in the present work.

### Conclusion.

We developed a two-part single-cell analysis strategy to assess the activities of AMPs. Method A permits measurement of the proliferation of single cells in the presence of various concentrations of an AMP, while method B assesses the death of individual bacterial cells following interaction with an AMP for a specific interval of time. This analysis enables determination of the number of the cells that are generated from single cells and the distribution of these numbers, providing information on the time course of the proliferation and death of single cells following interaction with AMPs; these results provide data that cannot be obtained using conventional methods (see details in section S3). In method A, the minimum concentration where *P*_single_ becomes 1 corresponds to the MIC. To elucidate the interaction time of AMP with bacterial cells required for bactericidal activity, we used method B. The *P*_single_ value obtained using method B represents the fraction of dead cells. For the interaction of Mag with E. coli cells, *P*_single_ increased with time, reaching ~1 for a 10-min interaction with 25 μM Mag. This result indicates that 25 μM Mag exhibits bactericidal activity against E. coli cells in less than 10 min; thus, for Mag, a short interaction time is sufficient to induce bacterial cell death.

## MATERIALS AND METHODS

### Materials.

Poly-l-lysine was purchased from Sigma-Aldrich Co. (St. Louis, MO). EZ rich defined medium (EZ medium) was purchased from TEKnova (Hollister, CA).

Mag (amino acid sequence GIGKFLHSAKKFGKAFVGEIMNS-NH_2_) was synthesized using an Initiator+ Alstra instrument (Biotage, Uppsala, Sweden) (32) and purified using reverse-phase high-performance liquid chromatography (HPLC) (29).

### MIC measurement.

The MIC values of Mag and tetracycline against E. coli growing in EZ medium were measured using the standard method (24, 46, 47). Briefly, an E. coli K-12 (NBRC 3301) suspension was subcultured on nutrient agar plates incubated overnight at 37°C to obtain single colonies, and a single bacterial colony then was grown in nutrient broth medium at 37°C for 6 to 7 h (exponential-phase cells) (20). The bacterial suspension was diluted to obtain a final bacterial concentration of ~1 × 10^5^ CFU/mL and then pelleted by centrifugation (620 × *g*, 10 min). The resulting pellet was washed and resuspended in EZ medium and then mixed with various concentrations of Mag (or tetracycline) solution in the individual wells of a 96-well plate. The final density of bacteria in the wells was 5 × 10^5^ CFU/mL, and the final compound concentrations in the wells ranged from 1 to 60 μM Mag or 1.5 to 64 μM tetracycline. After incubation at 37°C for 20 h, the absorbance at 600 nm was measured using a multimode microplate reader (Varioskan LUX; Thermo Fisher Scientific Inc., Waltham, MA). The MIC was defined as the lowest concentration of peptide at which the absorbance remained unchanged.

### Observation of the proliferation of a single cell in a chamber.

First, we prepared a handmade microchamber as follows. We made a square hole (10 mm by 10 mm by 3 mm) in a silicon rubber sheet (30 mm by 20 mm and 3 mm thick) and then placed this silicon rubber sheet on a slide glass, producing a chamber (10 mm by 10 mm by 3 mm). An aliquot (300 μL) of molten EZ medium containing 1.5% (wt/vol) agar was dispensed into the hole. After solidification, 10 μL of a suspension of E. coli in EZ medium (prepared as described above) was spread onto the agar. We controlled the density of the E. coli suspension so that all cells were separated on the agar medium, with intercellular distances of at least ~50 μm. Under these conditions, after incubation at 37°C, the distance between the centers of the resulting microcolonies was greater than ~50 μm. The chamber then was covered with a coverslip (18 mm by 18 mm). We incubated the E. coli cells (~1 × 10^4^ CFU/mL) in the chamber at 37°C for a specific time and then observed the cells at 25°C using the differential interference contrast (DIC) mode of a confocal laser scanning microscope (FV-1000; Olympus, Tokyo, Japan) or that of an inverse fluorescence DIC microscope (IX71; Olympus). To record all microcolonies, we scanned the agar surface of the microchamber as follows. An *xy* orthogonal coordinate system was defined on the agar surface. First, we focused on the leftmost part of the upper portion of the agar surface and scanned the agar surface along the *x* axis, i.e., we observed and recorded the rectangular zone with a vertical length (*h*) of the agar surface on the monitor of the computer (where *h* is the same as the vertical length of the monitor) from left to right. Next, we shifted the agar surface along the *y* axis by *h* and then scanned along the *x* axis in the opposite direction. We repeated this procedure to scan the total area of the agar surface. Next, we manually counted the cell numbers for each microcolony based on the images in the recorded video. This method provides an accurate count of the cell numbers of each microcolony, and thus, the potential overlap of recording and counting of microcolonies is eliminated.

### Single-cell analysis of the antimicrobial activity and bactericidal activity of Mag.

The single-cell analysis comprised two methods. In both methods, we adjusted the initial number of cells (same as the number of microcolonies) in a chamber so that there were 100 to 200 microcolonies per chamber (in special cases, per two chambers) and analyzed all the microcolonies.

Method A provided information on the antimicrobial activity of AMPs and antibiotics, yielding a value corresponding to the MIC. To examine the effect of AMPs (or antibiotics), we mixed an E. coli suspension (final cell density, ~1 × 10^4^ CFU/mL) in EZ medium with various concentrations of Mag (or tetracycline) solution (in EZ medium). Immediately after mixing, a 10-μL aliquot was transferred to the agar medium in a chamber and covered with a coverslip. We incubated the E. coli cells in the chamber at 37°C for a specific time (typically 3 h) and then observed the cells using DIC mode.

Method B provided information on the bactericidal activity of an AMP. First, we combined a cell suspension (final cell density, ~1 × 10^5^ CFU/mL) in EZ medium with Mag (or tetracycline) solution in EZ medium and incubated the mixture at room temperature (20 to 25°C). After a specific incubation time, an aliquot was taken from the suspension and diluted (10-fold for Mag, 40-fold for tetracycline) with fresh EZ medium; this dilution rendered the final concentrations of Mag and tetracycline to levels below those required to induce their antimicrobial activities. A 10-μL aliquot was transferred to the agar medium in a chamber for the single-cell analysis, and the chamber was incubated at 37°C for 3 h; we then counted the number of cells per microcolony using DIC mode. For the assessment of tetracycline with method B, we used two chambers under the same condition, permitting screening of a total of 100 to 200 microcolonies.

### Time-kill assay to measure the bactericidal activity of Mag.

An E. coli cell suspension (final cell density, 1.1 × 10^6^ CFU/mL) in EZ medium was combined with various concentrations of Mag (final concentrations, 13 and 25 μM) in a 5-mL microtube, and the mixture then was incubated at room temperature (20 to 25°C). At various time points during incubation, an aliquot was taken from the suspension and diluted (10-fold for experiments with Mag, 100-fold for experiments without Mag [due to the higher cell density]) with fresh EZ medium; an aliquot of 40 μL of the resulting suspension then was spread onto an agar plate in a petri dish. After a 24-h incubation at 37°C, we counted the number of bacterial colonies to determine the CFU/mL. Each measurement was performed three times using two replicates. The resulting values were used to calculate the mean and SD of the fraction of viable cells.

### Supporting information.

This article contains supporting information in the supplemental material addressing the following topics: theoretical distribution of the number of cells per microcolony during the proliferation of single cells, and the advantages of the single-cell analysis.
