# Supplementary material for: Single-Cell Analysis of the Antimicrobial and Bactericidal Activities of the Antimicrobial Peptide Magainin 2
Source: Microbiol Spectr. 2022 Jul 13;10(4):e00114-22. doi: 10.1128/spectrum.00114-22 (PMC9431230; doi:10.1128/spectrum.00114-22)
Supplement: Supplemental file 1 — Supplemental material. Download spectrum.00114-22-s0001.pdf, PDF file, 0.1 MB [file spectrum.00114-22-s0001.pdf]

Supplemental Material

Single-cell analysis for antimicrobial and bactericidal activities of antimicrobial peptide magainin 2

Farzana Hossain,[a] Md. Masum Billah,[b] and Masahito Yamazaki[a, b, c,*]

[a] Nanomaterials Research Division, Research Institute of Electronics, Shizuoka University, Shizuoka 422-8529, Japan, [b] Integrated Bioscience Section, Graduate School of Science and Technology, Shizuoka University, Shizuoka 422-8529, Japan, [c] Department of Physics, Faculty of Science, Shizuoka University, Shizuoka 422-8529, Japan.

S1. Theoretical distribution of the number of cells per microcolony proliferated from single cells

We considered proliferation patterns of single bacterial cells on agar in a microchamber. After several hours of incubation, long enough to achieve cell doubling, single cells proliferated into microcolonies, which are defined as aggregations of numerous cells originating from a single mother cell. Here, we demonstrate the theoretical distribution of cells per microcolony for proliferation from single cells based on a simple model.

At the start of incubation (t = 0), all microcolonies on agar have only a single cell, and there are N single cells are well separated on agar. We assume that all cells have the same generation time, τ, which we approximate to be 40 min. We group all single cells into four age brackets, each having an equal number of cells, at t = 0: A: 30–40 min old, B: 20–30 min old, C: 10–20 min old, and D: 0–10 min old. Then, we consider the proliferation of cells in each group after 1 h of incubation with each group completing cell doubling (each cell divides into 2 cells) in the following order: group A during the initial 10 min (t = 0–10 min), group B during the second 10 min (t = 10–20 min), group C during the third 10 min (t = 20–30 min), group D during the fourth 10 min (t = 30–40 min), group A for a second time during the fifth 10 min (t = 40–50 min), which results in microcolonies with 4 cells, and group B for the second time during the final 10 min (t = 50–60 min), which results in microcolonies with 4 cells. Hence, after 1 h incubation, the number of cells in microcolonies formed from single cells belonging to the groups A and B becomes 4, and the number of cells in the microcolonies formed from single cells belonging to the groups C and D becomes 2 (Fig. S1). These calculations are repeated to generate the theoretical distribution of microcolonies by number of cells.

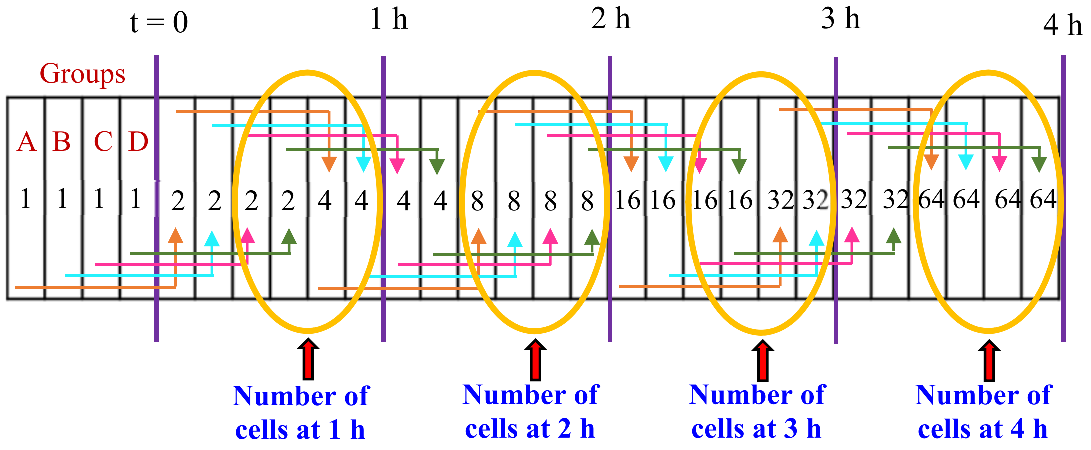

Figure S1. Schematic of the simple model of the proliferation of bacterial cells as explained in the text.

Considering the proportion of microcolonies by number of cells after 1 h of incubation, 50% have 2 cells and 50% have 4 cells (Fig. S2). After 2 h of incubation, all microcolonies have 8 cells (100%), after 3 h of incubation, the number of microcolony is evenly split between having 16 cells (50%) and 32 cells (50%), and after 4 h of incubation, all microcolonies have 64 cells (100%).

Figure S2.

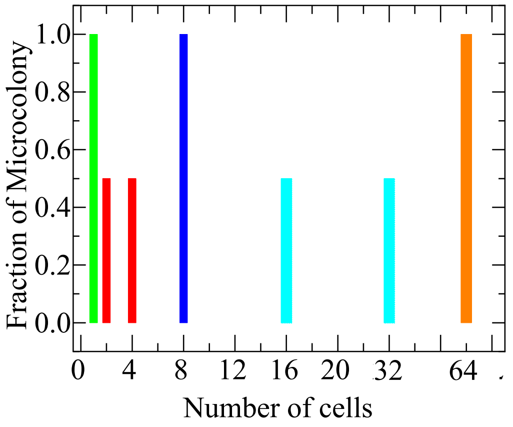

Figure S2. Theoretical distribution of fraction of microcolonies with given number of cells per microcolony after various incubation times: 0 h (green), 1 h (red), 2 h (blue), 3 h (cyan), and 4 h (orange).

S2. The MIC values determined by the single-cell analysis

The small difference between the MIC values obtained by the single-cell analysis and those by the standard MIC measurement may be due to the difference in the condition of cells. In the standard MIC measurement, cell suspensions are used for the interaction of AMPs and antibiotics with cells and thus all surroundings of the cells are the medium containing these compounds. On the other hand, in the single-cell analysis, single cells on agar plate are used for the interaction of AMPs and antibiotics with cells, and thus, a half of surrounding of the cells is the medium containing these compounds and the other half of surrounding of the cells is the agar plate. Therefore, the frequency of the interaction between the cells and AMPs and antibiotics on the agar plate becomes lower than that in the suspension. Moreover, the attachment of cells on the agar plate may increase the stability of cells, which may decrease susceptibility against AMPs and antibiotics.

S3. The advantages of the single-cell analysis.

Using the single-cell analysis, we can measure the number of the cells in a microcolony proliferated from a single cell and the distribution of the cell number per microcolony generated from many single cells as a function

of incubation time. Due to this characteristic, the single-cell analysis provides new information of the time course of the proliferation and the death of single bacterial cells interacting with AMPs for various interaction times, which cannot be obtained by the conventional methods, whereas the fraction of viable cells can be obtained by the standard time-kill assay. The new information obtained only by the single-cell analysis can be summarized as follows.

(1) In the growth curve of analysis, we can obtain the distribution of cell number per microcolony after the incubation of single cells for a specific time (Fig. 1G), providing the information on the proliferation of each single cell. The distribution of the number of cells proliferated from single cells (Fig. 1F) indicates the evidence of the fluctuation of the generation time of bacterial cells. In contrast, the standard growth curve of bacterial cells in suspension obtained by absorbance (turbidity) measurements (Fig. 1H) provides the average proliferation of all bacterial cells in a suspension, and thus, the information on the fluctuation of growth curve of each cell cannot be obtained.

(2) In Method A to investigate the effects of AMPs on the proliferation of single bacterial cells, we can obtain the fraction of microcolonies containing only a single cell, P_{single} (Fig. 2B), which reveals the fraction of cells that stop proliferating within the first-generation time among all initial cells. The minimum concentration to reach a P_{single} of 1 determined by the single-cell analysis corresponds to the MIC determined by the standard method. The distribution of cell number per microcolony in the various concentrations of AMPs (Fig. 2A) provides the information of the proliferation of single cells in the presence of AMPs. In contrast, the standard MIC measurement provides the average proliferation of all bacterial cells in the presence of AMPs and antibiotics in a suspension after 24 h incubation, and cannot indicate when the proliferation of cells stops.

(3) In Method B to investigate the effects of AMPs on the death of single bacterial cells, we can obtain the fraction of dead cells, P_{single}, (Fig. 3B) after the interaction of AMPs with cells for a specific time, revealing the interaction time required for their bactericidal activity. As described in the main text, the single-cell analysis is a higher sensitive method to detect live cells than the time-kill assay.

Therefore, these results well demonstrate that the single-cell analysis provides various useful information on the antimicrobial and bactericidal activities of AMPs at the single cell level.

Another advantage of the single-cell analysis is that the results are obtained more rapidly (3 h) compared with the conventional methods (the MIC method, the MBC method, and the time-kill assay) (24 h).
